# Hearing loss and risk of major osteoporotic fracture: a population-based cohort study in the United Kingdom

**DOI:** 10.1007/s11657-024-01484-2

**Published:** 2025-01-28

**Authors:** Sara Khalid, Daniel Prieto Alhambra, Seyed Alireza Hasheminasab, Yana Vinogradova, Nadeem Qureshi, Michaela Ratzinger, Vanessa Brunetti, Adrian Salas, Laura Canals

**Affiliations:** 1 Oxford University, Oxford, UK; 2 University of Nottingham, Nottingham, UK; 3 Amgen Inc., Italia 415, 2Nd Floor - Vicente Lopez (1368), Buenos Aires, Argentina

**Keywords:** Hearing loss, Hearing impairment, Osteoporosis, Major osteoporotic fracture, Hip fracture, Risk factor

## Abstract

***Summary*:**

Using the UK Clinical Practice Research Datalink, our cohort study matched 237,297 individuals with hearing loss (HL) to 829,431 without HL. The study found an 8–10% higher risk of major osteoporotic fracture in individuals with HL compared to those without. Additionally, within the HL cohort, we identified risk factors for potential inclusion in fracture risk models.

**Purpose:**

Assess association between hearing loss (HL) and major osteoporotic fracture (MOF; spine, wrist/forearm, shoulder/proximal humerus, hip) in individuals aged ≥ 60 years, and risk factors for MOF in individuals with HL.

**Methods:**

From the UK Clinical Practice Research Datalink, our cohort study matched individuals aged ≥ 60 years diagnosed with HL (READ/ICD-10 codes; 01January2001–31December2021; index event), without secondary osteoporosis causes, with up to five individuals without HL (birth, index year, sex, general practice). Incidence rates and Cox proportional hazard ratios (HL vs. no HL; stratified by low/high fracture risk) were calculated for MOF and hip fracture; multivariate logistic regression assessed risk factors for MOF and hip fracture (HL cohort).

**Results:**

A total of 237,297 individuals with HL matched to 829,431 without HL, with a median age of 74 and 72 years, respectively. Compared with those without HL, individuals with HL had greater frailty (severe electronic frailty index, 5.9% vs. 2.7%), higher incidence of prior falls (14.1% vs. 10.6%), longer mean follow-up with higher incidence of MOF and hip fractures (5.1 vs. 4.4 years, 20.1 and 5.32 vs. 16.58 and 4.54 per 1000 person-years, respectively) and higher risk of MOF and hip fracture (adjusted HR, 1.10 and 1.08, respectively). Significant risk factors for MOF and hip fracture included age ≥ 70 years, fracture history, falls, osteoporosis diagnosis, chronic obstructive pulmonary disorder and cardiovascular disease (HL cohort).

**Conclusion:**

In individuals with HL, we observed an 8–10% higher risk of MOF and hip fracture versus individuals without HL and identified risk factors for potential inclusion in fracture risk models.

**Supplementary Information:**

The online version contains supplementary material available at 10.1007/s11657-024-01484-2.

## Introduction

The World Health Organization (WHO) defines hearing loss (HL) as the inability to hear below 20 dB in one or both ears [1]. Age is known to be one of the main causes of HL and its prevalence increases with age [2, 3]. HL affects 1.57 billion people worldwide, approximately 20% of the global population, with most of them (62%) older than 50 years [4]. In the UK, HL affects approximately 11 million people, with 71% aged 60 or over [5, 6]. HL is a public health issue, being the third most common global cause of years lived with disability, and is associated with comorbidities including arthritis and cardiovascular disease, increased disease burden and poor health in individuals aged 65 and older [3, 4, 7]. Moreover, global prevalence of HL has increased by approximately 80% in the last 30 years and is estimated to increase by 56% by 2050 [4].

Osteoporosis (OP) is a bone disorder characterised by low bone mineral density and decreased bone strength, which increases the risk of fragility fractures [8, 9]. Over 27.5 million people in Europe [10] and almost 3.8 million in the UK are estimated to have OP [11]. The incidence of OP is higher in women than in men, especially after menopause [12]. Moreover, the incidence of OP increases with age, with OP being the leading cause of bone fracture in older people [9] and being associated with increased risks of hip and vertebral fractures, short-term mortality and high medical costs [8, 9].

Previous studies have reported OP to be a risk factor for HL, which might play a role in age-related HL [13–16]. Alterations of bone mass, density and cushioning of the middle ear’s mechanics observed in osteopenic and osteoporotic patients may lead to conductive HL. Reduction of bone mineral density might cause changes in the transmission characteristics of the bone, altering the functioning of the middle ear [17, 18]. Moreover, osteoporotic bone metabolism alters the calcium ionic endolymphatic flow of the cochlea, which may disturb the mechanoelectrical transduction of the cochlea [19]. HL has been also associated with a statistically significant increase in the risk of falls in older adults [3, 20, 21]. In turn, falls increase the risk of fracture [22], which can have a significant impact on a patient’s quality of life [23]. Established tools to predict the risk of major osteoporotic fracture (MOF; clinical spine, wrist/forearm, shoulder/proximal humerus, hip), such as FRAX [24] and QFracture™ [25], were developed for the general and osteoporotic population and are widely used in clinical practice. However, these tools need to be improved to include novel risk factors to be applied in special population [26, 27].

Our study assessed (1) the association between HL and risk of MOF in individuals aged ≥ 60 years and (2) potential risk factors associated with 1- and 10-year risk of MOF and hip fractures in patients with HL, and combined key predictors of fracture risk to derive major osteoporotic prediction tools in the HL population.

## Methods

The study methods are summarised below; further details are provided in the Supplementary material.

### Study design

This matched cohort study used secondary health data from the UK Clinical Practice Research Datalink (CPRD-GOLD) [28], which is linked to Hospital Episode Statistics (HES) [29], Admitted Patient Care (APC), indices of multiple deprivation (IMD) [30] and Office for National Statistics [10, 31]. CPRD GOLD is a database of anonymised medical records from general practitioners (GPs) in the UK, linked to HES, and provides data for over nine million patients from > 900 practices, which is representative of the UK general population. The IMD database provides data on the relative deprivation of small areas of the UK [30].

Access to CPRD data was subject to a protocol approval by the UK Independent Scientific Advisory Committee, a non-statutory expert advisory body established by the Secretary of State for Health to provide scientific advice on research requests to access data provided by CPRD. All data used in the CPRD were taken from anonymised electronic health records; no patients were identifiable. Patients had already consented for their data collected in CPRD to be used for analysis purposes; therefore, informed consent was not necessary for the current study.

### Study population

**Fig. 1:**
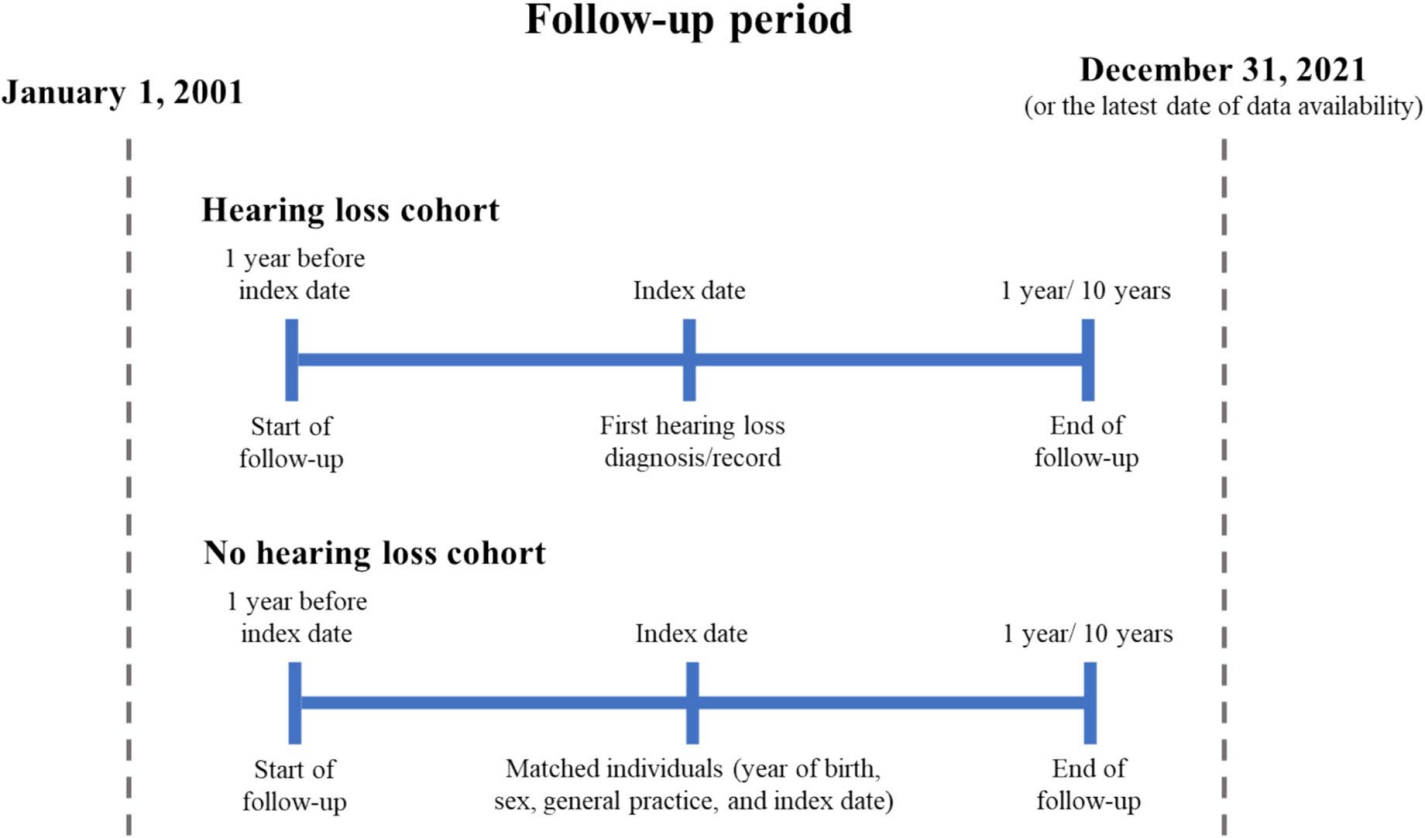
Study design schema

Our HL cohort included individuals aged ≥ 60 years with a diagnosis of HL between January 1, 2001 and December 31, 2021 (index event; identified using READ and ICD-10 diagnosis codes [Supplementary Table S1]) or the latest date of CPRD data availability (Fig. 1) and registered in CPRD for at least one year before their index date. In order to focus on the association between hearing loss and fracture risk, patients with history of vestibular dysfunction, osteomalacia and/or other secondary osteoporotic causes (endocrine disorders, Cushing syndrome, hyperparathyroidism, type 1 diabetes mellitus, chronic active hepatitis, pancreatic insufficiency, ankylosing spondylitis, multiple sclerosis, osteogenesis imperfecta or aromatase inhibitors use) were excluded.

For the non-HL cohort, each individual in the HL cohort was matched with up to five individuals with no diagnosis of HL, by year of birth, sex, general practice and index date (date of the first diagnosis/record of HL for their matched HL case). Individuals with less than 1 year of data before entering the non-HL cohort were excluded. Comparability after matching was assessed by checking the standardized mean difference of matched variables.

Individuals in both cohorts were followed from their index date until (1) MOF (clinical spine, wrist/forearm, shoulder/proximal humerus, hip; identified using READ and ICD-10 diagnosis codes [Appendix 1]); or (2) censoring at the earliest of death, migration/transfer out, practice last collection date, end of study period (based on data availability at extraction date) or end of follow-up period (1 or 10 years after index date).

### Study outcomes

Study outcomes were MOF and hip fracture during follow-up, risk factors included in the QFracture™ [25] prediction tool (https://qfracture.org/); other risk factors described in the literature (socio-economic status, occupation, family history and previous meningitis).

The following variables were summarised at index date: demographics (i.e. age, sex), lifestyle (i.e. smoking status, alcohol consumption, body-mass index), OP fracture risk (i.e. previous osteoporotic or non-osteoporotic fracture, OP diagnosis, use of bisphosphonates and other OP medications, family history of OP, history of falls, difficulty walking), comorbidities and individual medications received in the year prior to index date (Supplemental Material).

### Statistical analysis

For each cohort (HL, non-HL), baseline variables were described using summary statistics, and MOF incidence rates (IR) during follow-up calculated overall and by fracture type. The relative risk of MOF in the HL versus non-HL cohort was assessed using Cox proportional hazards ratios (HR) and 95% confidence intervals (CI), adjusted for baseline characteristics (sociodemographic, lifestyle, medical history, and medication use).

Within the HL cohort, potential risk factors for MOF were assessed using multivariable logistic regression; specifically, adjusted odds ratios (OR). Lasso regression was used to select predictors for the multivariable model and a prediction algorithm (logistic regression) trained on different imputation sets. Model discrimination was evaluated using area under the curve (AUC). Calibration was assessed using predicted/observed plots stratified by risk deciles, age (5-year), and sex. Survival analysis was used to predict the 10-year risk of MOF based on the initial set of predictor variables. Concordance index and Brier score were used to evaluate model performance. Concordance index measures the proportion of pairs of individuals where the model correctly predicts the order of survival times [32], while Brier score measures the overall accuracy of the model's predicted survival probabilities [33].

An analysis of the absolute and relative risk (HL vs. no HL) of overall MOF and of each major fracture subtypes, clustered by age, sex and general practice and stratified by high risk of fracture (prior history of fractures, OP diagnosis and/or anti-OP treatment) was conducted. Key risk factors were those considered statistically significant.

Missing data were handled using multiple imputation (*n* = 10) and combined using Rubin’s rules [34].

The statistical analysis was performed using the STAT 17 and R programming language. All *p* values < 0.05 were considered statistically significant.

## Results

### Patient baseline characteristics

Overall, 237,297 individuals with HL were matched to 829,431 individuals without HL. Baseline and OP-related characteristics are summarised in Table 1 (and Supplementary Table S2). Mean age and the distribution of men and women were similar across individuals with and without HL (74.1 vs. 72.5 years, and 52%/48% vs. 53%/47%, respectively; Table 1). Compared to individuals without HL, those with HL were frailer (Electronic Frailty index of mild to severe, 29.2% vs. 17.3%), had a higher comorbidity burden (Charlson comorbidity score of moderate or severe, 23.3% vs. 19.8%) and were more likely to be prescribed systemic steroids (43% vs. 32%) and proton-pump inhibitors (31% vs. 24%) in the year prior to index event (Table 1 and Supplementary Table S2).

**Table 1:** Baseline and OP-related characteristics of individuals with and without HL

	HL *N* = 237, 297	No HL *N* = 829, 431
Baseline characteristics		
Sex, *n* (%)		
Women	113,166 (47.7)	386,161 (46.6)
Men	124,131 (52.3)	443,270 (53.4)
Age (years)		
Mean (SD)	74.1 (8.7)	72.5 (8.3)
Median (Q1–Q3)	74 (67–81)	72 (66–79)
60–69 years, *n* (%)	82,104 (34.6)	347,551 (41.9)
70–79 years, *n* (%)	87,706 (37.0)	298,340 (36.0)
80–89 years, *n* (%)	57,544 (24.2)	161,897 (19.5)
> 90 years, *n* (%)	9943 (4.2)	21,643 (2.6)
Electronic frailty index, *n* (%)		
Fit	168,061 (70.8)	685,962 (82.7)
Mild	55,142 (23.2)	120,504 (14.5)
Moderate	12,134 (5.1)	20,311 (2.4)
Severe	1960 (0.8)	2654 (0.3)
Charlson comorbidity score, *n* (%)		
None	89,676 (37.8)	363,950 (43.9)
Mild	92,334 (38.9)	301,590 (36.4)
Moderate	39,588 (16.7)	118,661 (14.3)
Severe	15,699 (6.6)	45,230 (5.5)
OP-related characteristics		
High risk of fracture, *n* (%)	53,805 (22.7)	165,987 (20.0)
Diagnosis of osteoporosis	15,357 (6.5)	42,033 (5.1)
Use of bisphosphonates	15,896 (6.7)	42,952 (5.2)
Previous osteoporotic fracture	40,972 (17.3)	130,332 (15.7)
Femur	767 (0.3)	2704 (0.3)
Hip	3972 (1.7)	13,985 (1.7)
Pelvic	773 (0.3)	2237 (0.3)
Shoulder	914 (0.4)	3040 (0.4)
Spine	1861 (0.8)	5244 (0.6)
Tibia	1919 (0.8)	6629 (0.8)
Wrist	10,691 (4.5)	33,399 (4.0)
Other	20,075 (8.5)	63,094 (7.6)
Previous non-osteoporotic fractures, *n* (%)	20,113 (8.5)	64,038 (7.7)
Family history of osteoporosis, *n* (%)	1184 (0.5)	3345 (0.4)
History of falls, *n* (%)	33,443 (14.1)	88,296 (10.6)
Difficulty walking, *n* (%)	5155 (2.2)	11,908 (1.4)

*HL* hearing loss, *OP* osteoporosis, *Q* quartile, *SD* standard deviation

Regarding the OP-related characteristics, a similar percentage of individuals with and without HL were classified as being at high risk of osteoporotic fracture (22.7% and 20.0%, respectively) and had experienced at least one osteoporotic fracture (17.3% and 15.7%), most commonly wrist fractures (4.5% and 5.0%). Similar percentages were also observed between the two groups for non-OP fractures (8.5% vs. 7.7%), family history of OP (0.5% vs. 0.4%) and difficulty walking (2.2% vs. 1.4%). However, individuals with HL were more likely to have a history of falls (14.1% vs. 10.6%) (Table 1).

### HL and MOF

MOF during follow-up are summarised in Table 2. Individuals with HL had longer mean follow-up (5.1 vs. 4.4 years) and were more likely to experience MOF (incidence, 10.2% vs. 7.3%) than individuals without HL (data for MOF are summarised in Supplementary Table S3).

**Table 2:** Incidence rate of MOF during follow-up in individuals with and without HL

	HL *N* = 237, 297	No HL *N* = 829, 431
Incidence rate (95% CI) per 1000 person-years	20.11 (19.86, 20.36)	16.58 (16.44, 16.72)
Hip	5.32 (5.19, 5.45)	4.54 (4.47, 4.61)
Pelvic	0.78 (0.73, 0.83)	0.60 (0.58, 0.63)
Shoulder	0.81 (0.76, 0.86)	0.68 (0.66, 0.71)
Spine	1.92 (1.84, 2.00)	1.40 (1.37, 1.44)
Wrist	3.58 (3.47, 3.68)	2.97 (2.91, 3.03)
Other	7.71 (7.55, 7.87)	6.38 (6.29, 6.46)

*CI* confidence interval, *HL* hearing loss

The incidence rate (95% CI) of MOF was 20.1 (19.86, 20.36) per 1000 person-years in the HL cohort vs. 16.6 (16.44, 16.72) in the non-HL cohort; hip fracture, 5.32 (5.19, 5.45) per 1000 person-years in the HL cohort vs. 4.54 (4.47, 4.61) in the non-HL cohort.

Cox proportional hazards ratios, clustered by matched set (containing data for age, sex and general practice) and stratified by high fracture risk (yes/no) estimated an 18% increased risk of MOF in the HL cohort vs. non HL cohort (HR [95% CI] 1.18 [1.16, 1.20]), falling to 10% when further adjusted by lifestyle, morbidities and medications (1.10 [1.08, 1.12]). This increased risk was observed across all fracture types (all *p* values < 0.001), with the greatest increase observed for spine fractures (1.17 [1.11, 1.23]), followed by pelvic (1.11 [1.03, 1.20]), wrist (1.11 [1.07, 1.15]) and shoulder (1.09 [1.01, 1.17]), and the smallest increase observed for hip fracture (1.08 [1.05, 1.11]).

### Risk factors associated with 1- and 10-year fracture risk (HL cohort)

**Fig. 2:**
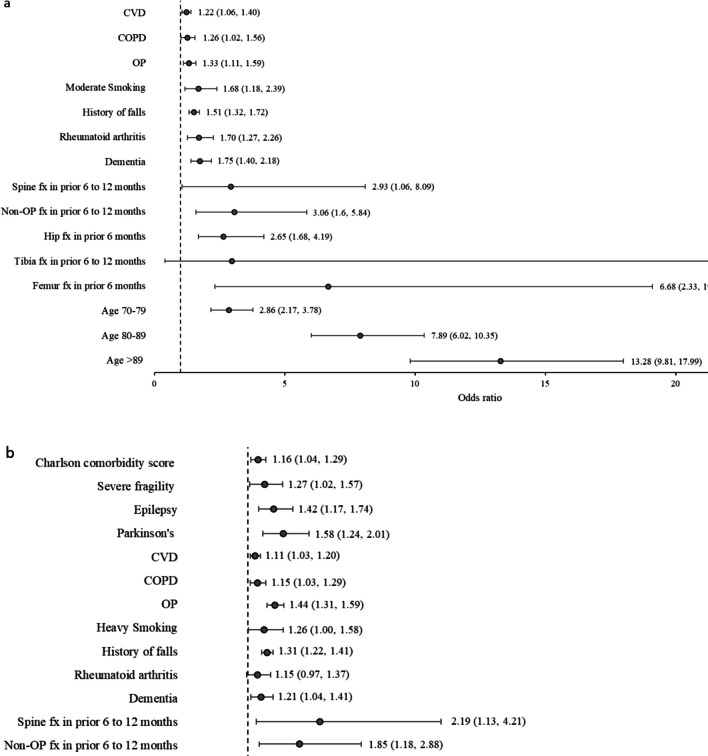
Key risk factors for 1-year hip fracture **a** and MOF **b**. *COPD* chronic obstructive pulmonary disease, *CVD* cardiovascular disease, *Fx* fracture, *OP* osteoporosis

The multivariate regression analysis of the HL cohort is summarised in Fig. 2 (1-year risk). Significant 1-year risk factors for both hip and MOF were age ≥ 70 years (vs. age < 70), with the largest increase in risk observed in individuals > 89 years (OR [95% CI]: hip, 13.28 [9.81, 17.09]; MOF, 2.85 [2.51, 3.25]), with history of fractures and falls (OR: hip, 1.51–6.68; MOF, 1.31–3.42), and dementia (OR [95% CI]: hip, 1.75 [1.40, 2.18]; MOF, 1.21 [1.04, 1.41]). Additional 1-year risk factors for hip fracture included cardiovascular disease (1.22 [1.06, 1.40], chronic pulmonary obstructive disease (1.26 [1.02, 1.56]) and rheumatoid arthritis (1.70 [1.27, 2.26]) (Fig. 2a). Additional 1-year risk factors for MOF included Parkinson’s disease (1.58 [1.24, 2.01]), epilepsy (1.42 [1.17, 1.74]), severe frailty (1.27 [1.02, 2.57]) and Charlson comorbidity score (1.16 [1.04, 1.29]) (Fig. 2b) (Supplementary Tables S4 and S5).

In general, 10-year risk factors for fractures were similar to 1-year risk factors, with the addition of blind loop syndrome: OR [95% CI], 4.05 [0.52, 31.62] and 3.42 [1.07, 10.98] for hip fracture and MOF, respectively, although the former was not statistically significant (Supplementary Tables S4 and S5).

### Fracture risk factors (HL cohort)

Lasso regression models identified age, previous fractures, and OP as significant predictors of both hip- and MOF at 1-year. Overall, individuals aged ≥ 70 years showed a higher risk for hip fractures in the logistic regression model compared with those aged 60–69 years (Table 3 and Supplementary Table S6).

**Table 3:** Logistic regression model estimates for 1-year hip and MOF risk in patients with HL

	Hip fracture		MOF	
	OR	*p* value	OR	*p* value
Age > 89 years	2.63	< 0.0001	1.06	< 0.0001
Age 80–89 years	2.08	< 0.0001	0.75	< 0.0001
Age 70–79 years	1.09	< 0.0001	0.25	< 0.0001
Femur fracture in prior 12 months	0.77	0.03	0.29	0.2
Hip fracture in prior 12 months	0.44	0.003	0.52	< 0.0001
Osteoporosis	0.29	0.0009	0.41	< 0.0001

*MOF* major osteoporotic fracture, *OR* odds ratio

Good model discrimination and calibration was achieved: area under the curve (AUC), 0.811 (95% CI: 0.772, 0.850) and 0.733 (95% CI: 0.710, 0.755) (Supplementary Fig. S1) and calibration slope of 0.98 and 1.02 for the 1-year hip- and MOF models, respectively. AUC was reduced for the 10-year models.

For 10-year risk prediction, model performance of the time-to-event models was evaluated using concordance and brier score, with values of 0.736 and 0.133, respectively.

## Discussion

HL is a global health issue, especially among older individuals, and has been associated with an increased risk of falls and poor health in this population [4, 7, 21]. Our UK population-based study assessed the association between HL and MOF in individuals aged ≥ 60 years, and found individuals with HL to be at increased risk of hip-fracture and MOF compared with individuals without HL (i.e. normal hearing). In addition, we identified risk factors for 1- and 10-year hip- and MOF in this population, with age being a key risk factor. Over a median follow-up of 4.2 and 3.2 years in individuals with versus without HL, incidence rates of hip and MOF were 5.32 vs. 4.45 and 20.11 vs. 16.58 per 1000 person-years, respectively. Moreover, when adjusted for lifestyle, comorbidities and medications, individuals with HL had a statistically significant 10% increase in the risk of MOF compared with individuals without HL. Spine fractures were associated with the highest increase in risk (17%) and hip fractures associated with the lowest increase in risk (8%). Previous studies have also reported an association between HL and increased fracture risk. For example, compared with controls, Kim et al. reported higher risk of spine- (HR: 1.32) and hip (HR: 1.70) fractures in individuals aged ≥ 60 years. While this increase in risk is larger than observed in the current study, only patients with severe or profound hearing impairment were included in the study by Kim et al. [35].

We also assessed risk factors for 1- and 10-years hip fracture and MOF among individuals with HL, identifying fractures 6–12 months prior to HL diagnosis, history of OP and age ≥ 70 years old to be key risk factors. Our data align with prior studies reporting incidence of hip and other fracture types, including spine, wrist and distal femur, increases with age [36]. Thus, clinicians should be aware of these associations, encourage patients to check their hearing status and, if necessary, refer patients to other specialties for appropriate care. These results also suggest that interventions aimed at reducing the prevalence of aforementioned risk factors may help mitigate the risk of hip- and MOF.

The risk factors identified in our study were combined into prediction models for hip- and MOF showing that age, previous history of fractures, and OP were significant predictors. Furthermore, survival analysis indicated these models had comparable performance to existing tools, such as QFracture™ (AUC of 0.811 and 0.733 for the 1-year hip- and MOF models, respectively), while utilizing fewer variables and routinely available measurements. While we identified frailty and Charlson comorbidity score as risk factors for MOF, these variables are not currently included in tools such as QFracture™. Hence, our findings suggest that the identified factors and presented prediction models should be included in fracture risk assessment tools.

Our study has some limitations. Risk factors and outcomes of interest could have been misclassified in patient records, causing information bias. In addition, the data source used for the study is highly representative of the wider UK population. Therefore, the study population may be similar to the target HL population, limiting the representativity from the entire population to this subpopulation. We cannot rule out the possibility that the increased risk observed between HL and fracture risk might be due to individual’s age or frailty. Moreover, as suggested previously [21], hearing aids may modulate the risk of falls in patients with HL; therefore, as our dataset did not include information on hearing aid use, the effect of HL on fracture risk may be underestimated in our HL cohort. Despite these limitations, our HL and non-HL cohorts exceeded the minimum recommended sample size of patients per group indicating our study is robust and representative of the UK population with HL. Furthermore, when assessing the association of HL and fractures risk, confounding was addressed by matching each individual in the HL cohort with up to five individuals without HL by year of birth, sex, General Practice, and index date, and adjusting the association with other risk factors.

## Conclusion

Our population-based matched cohort study found individuals with HL to be at increased risk of hip- and MOF compared with individuals without HL. Among individuals with HL, prior fracture and OP, older age (≥ 70 years), and medical conditions including Parkinson’s disease, dementia and cardiovascular disease were identified as risk factors for fractures. In addition, we developed a model to predict fracture risk in this population.

## Supplementary Information

Below is the link to the electronic supplementary material.

Supplementary file1 (DOCX 195 KB)

Supplementary file2 (XLSX 41 KB)

## Data Availability

Qualified researchers may request data from Amgen clinical studies. Complete details are available at the following: https://wwwext.amgen.com/science/clinical-trials/clinicaldatatransparencypractices/clinical-trial-data-sharing-request.

## References

[1] World Health Organization. Deafness and hearing loss. [Available from: https://www.who.int/news-room/fact-sheets/detail/deafness-and-hearing-loss. Accessed 22 May 2024

[2] Jayakody DMP, Friedland PL, Martins RN, Sohrabi HR (2018) Impact of aging on the auditory system and related cognitive functions: a narrative review. Front Neurosci 12:125 29556173 10.3389/fnins.2018.00125 PMC5844959

[3] Li CM, Zhao G, Hoffman HJ, Town M, Themann CL (2018) Hearing disability prevalence and risk factors in two recent national surveys. Am J Prev Med 55(3):326–335 30031639 10.1016/j.amepre.2018.03.022 PMC11296353

[4] GBD 2019 (2021) Hearing Loss Collaborators. Hearing loss prevalence and years lived with disability, 1990–2019: findings from the Global Burden of Disease Study 2019. Lancet 397(10278):996–1009 10.1016/S0140-6736(21)00516-X PMC7960691 33714390

[5] British Academy of Audiology. Hearing loss and deafness. [Available from: https://www.baaudiology.org/about/media-centre/facts-about-hearing-loss-and-deafness/. Accessed 22 May 2024

[6] Lee JW, Bance ML (2019) Hearing loss. Pract Neurol 19(1):28–35 30185631 10.1136/practneurol-2018-001926

[7] McKee MM, Stransky ML, Reichard A (2018) Hearing loss and associated medical conditions among individuals 65 years and older. Disabil Health J 11(1):122–125 28596096 10.1016/j.dhjo.2017.05.007

[8] Compston JE, McClung MR, Leslie WD (2019) Osteoporosis Lancet 393(10169):364–376 30696576 10.1016/S0140-6736(18)32112-3

[9] Romano F, Serpico D, Cantelli M, Di Sarno A, Dalia C, Arianna R et al (2023) Osteoporosis and dermatoporosis: a review on the role of vitamin D. Front Endocrinol (Lausanne) 14:1231580 37693364 10.3389/fendo.2023.1231580 PMC10484397

[10] Svedbom A, Hernlund E, Ivergård M, Compston J, Cooper C, Stenmark J et al (2013) Osteoporosis in the European Union: a compendium of country-specific reports. Arch Osteoporos 8(1):137 24113838 10.1007/s11657-013-0137-0 PMC3880492

[11] Willers C, Norton N, Harvey NC, Jacobson T, Johansson H, Lorentzon M et al (2022) Osteoporosis in Europe: a compendium of country-specific reports. Arch Osteoporos 17(1):23 35079919 10.1007/s11657-021-00969-8 PMC8789736

[12] Salari N, Ghasemi H, Mohammadi L, Behzadi MH, Rabieenia E, Shohaimi S et al (2021) The global prevalence of osteoporosis in the world: a comprehensive systematic review and meta-analysis. J Orthop Surg Res 16(1):609 34657598 10.1186/s13018-021-02772-0 PMC8522202

[13] Yeh MC, Weng SF, Shen YC, Chou CW, Yang CY, Wang JJ et al (2015) Increased risk of sudden sensorineural hearing loss in patients with osteoporosis: a population-based, propensity score-matched, longitudinal follow-up study. J Clin Endocrinol Metab 100(6):2413–2419 25879512 10.1210/jc.2014-4316

[14] Upala S, Rattanawong P, Vutthikraivit W, Sanguankeo A (2017) Significant association between osteoporosis and hearing loss: a systematic review and meta-analysis. Braz J Otorhinolaryngol 83(6):646–652 27670202 10.1016/j.bjorl.2016.08.012 PMC9449069

[15] Yoo JI, Park KS, Seo SH, Park HW (2020) Osteoporosis and hearing loss: findings from the Korea National Health and Nutrition Examination Survey 2009–2011. Braz J Otorhinolaryngol 86(3):332–338 30827872 10.1016/j.bjorl.2018.12.009 PMC9422524

[16] Gargeshwari A, Singh NK, Kumar P, Jha RH (2017) Effect of lowered bone mineral density on the outcomes of audiological tests: a preliminary study. J Indian Speech Lang Hear Assoc 31(1):29–35

[17] Singh NK, Jha RH, Gargeshwari A, Kumar P (2018) Altered auditory and vestibular functioning in individuals with low bone mineral density: a systematic review. Eur Arch Otorhinolaryngol 275(1):1–10 29043479 10.1007/s00405-017-4768-4

[18] Kumar P, Singh NK, Gargeshwari A, Jha RSR (2019) Changes in middle ear transmission characteristics secondary to altered bone remodelling. Osteoporos Int 30(4):863–70 30652218 10.1007/s00198-019-04834-w

[19] Kim SY, Kong IG, Lim H, Choi HG (2018) Increased Risk of Sudden Sensory Neural Hearing Loss in Osteoporosis: A Longitudinal Follow-Up Study. J Clin Endocrinol Metab 103(8):3103–3109 29846624 10.1210/jc.2018-00717

[20] Jiam NT, Li C, Agrawal Y (2016) Hearing loss and falls: a systematic review and meta-analysis. Laryngoscope 126(11):2587–2596 27010669 10.1002/lary.25927

[21] Tiase VL, Tang K, Vawdrey DK, Raso R, Adelman JS, Yu SP et al (2020) Impact of hearing loss on patient falls in the inpatient setting. Am J Prev Med 58(6):839–844 32444002 10.1016/j.amepre.2020.01.019

[22] Berry SD, Miller RR (2008) Falls: epidemiology, pathophysiology, and relationship to fracture. Curr Osteoporos Rep 6(4):149–154 19032925 10.1007/s11914-008-0026-4 PMC2793090

[23] Palacios S, Neyro JL, Fernández de Cabo S, Chaves J, Rejas J (2014) Impact of osteoporosis and bone fracture on health-related quality of life in postmenopausal women. Climacteric 17(1):60–70 23710562 10.3109/13697137.2013.808182

[24] Kanis JA, Johnell O, Oden A, Johansson H, McCloskey E (2008) FRAX and the assessment of fracture probability in men and women from the UK. Osteoporos Int 19(4):385–397 18292978 10.1007/s00198-007-0543-5 PMC2267485

[25] Hippisley-Cox J, Coupland C (2009) Predicting risk of osteoporotic fracture in men and women in England and Wales: prospective derivation and validation of QFractureScores. BMJ 339:b4229 19926696 10.1136/bmj.b4229 PMC2779855

[26] Livingstone SJ, Morales DR, McMinn M, Eke C, Donnan P, Guthrie B (2022) Effect of competing mortality risks on predictive performance of the QFracture risk prediction tool for major osteoporotic fracture and hip fracture: external validation cohort study in a UK primary care population. BMJ Med 1(1):e000316 36936595 10.1136/bmjmed-2022-000316 PMC9978756

[27] Vandenput L, Johansson H, McCloskey EV, Liu E, Åkesson KE, Anderson FA et al (2022) Update of the fracture risk prediction tool FRAX: a systematic review of potential cohorts and analysis plan. Osteoporos Int 33(10):2103–2136 35639106 10.1007/s00198-022-06435-6

[28] Clinical Practice Research Datalink (CPRD) [Available from: https://cprd.com. Accessed 22 May 2024

[29] Hospital Episode Statistics (HES) [Available from: https://digital.nhs.uk/data-and-information/data-tools-and-services/data-services/hospital-episode-statistics. Accessed 22 May 2024

[30] Index of Multiple Deprivation (IMD) [Available from: https://data.cdrc.ac.uk/dataset/index-multiple-deprivation-imd. Accessed 22 May 2024

[31] Office for National Statistics [Available from: https://www.ons.gov.uk/. Accessed 22 May 2024

[32] Harrell FE Jr, Lee KL, Mark DB (1996) Multivariable prognostic models: issues in developing models, evaluating assumptions and adequacy, and measuring and reducing errors. Stat Med 15(4):361–387 8668867 10.1002/(SICI)1097-0258(19960229)15:4<361::AID-SIM168>3.0.CO;2-4

[33] Brier GW (1950) Verification of forecasts expressed in terms of probability. Mon Weather Rev 78:1–3

[34] Rubin DB (1987) Underlying Bayesian theory. Multiple imputation for nonresponse in surveys. 1–76. 10.1002/9780470316696

[35] Kim SY, Lee JK, Sim S, Choi HG (2018) Hearing impairment increases the risk of distal radius, hip, and spine fractures: a longitudinal follow-up study using a national sample cohort. PLoS ONE 13(2):e0192820 29438391 10.1371/journal.pone.0192820 PMC5811044

[36] Ensrud KE (2013) Epidemiology of fracture risk with advancing age. J Gerontol A Biol Sci Med Sci 68(10):1236–1242 23833201 10.1093/gerona/glt092

