# Supplementary material for: Hearing loss and risk of major osteoporotic fracture: a population-based cohort study in the United Kingdom
Source: Arch Osteoporos. 2025 Jan 28;20(1):14. doi: 10.1007/s11657-024-01484-2 (PMC11774978; doi:10.1007/s11657-024-01484-2)
Supplement: Supplementary file 1 — Supplementary file1 (DOCX 195 KB) [file 11657_2024_1484_MOESM1_ESM.docx]

**SUPPLEMENTARY METHODS**

**Sample size calculation**

For the relative risk analysis, a sample of 10,926 participants in the HL cohort and 54,630 in the non-HL cohort would be 90% powered and would suffice to find as statistically significant (p-value < 0.05) a Hazard Ratio (HR) of 1.10 or upper. The probability of the event was expected to be 0.2 in both cohorts.

For the predicting model, current guidelines suggest that a minimum of 100 patients with the outcome of interest and 100 patients without the event of interest are required to provide reliable estimates of model performance [1]. Considering that it was estimated that the general population in the UK underwent 3.6 fractures per 100 people per year, eight million people aged 60 years and older had been diagnosed with HL or impairment in the UK [2, 3], and assuming higher differences from HL/impairment patients to the general population, it was anticipated to exceed the number needed according to these guidelines.

**Risk factors**

- Demographics
  - ethnicity (White, Black, Asian, other, not recorded)
  - age and sex as matching variables
  - social deprivation in quintiles for linked practices (from IMD data)
  - care or nursing home status
- Life style
  - smoking status with the following categories: non-smoker, ex-smoker, light smoker (<10 cigarettes/day), moderate smoker (10-19 cigarettes per day), heavy smoker (20 or more cigarettes per day)
  - Alcohol, with the following categories: none, trivial (<1 unit/day), light (1-2 units/day), medium (3-6 units/day), heavy (7-9 units/day), very heavy (>9 units/day)
  - Body-mass index
- Osteoporosis and fracture related covariates
  - any previous osteoporotic fracture
  - any previous non-osteoporotic fracture
  - diagnosis of osteoporosis
  - use of bisphosphonates (and other osteoporosis medications)
  - family history of osteoporosis
  - history of fall
  - difficulty walking
- Morbidities
  - anxiety
  - asthma
  - any cancer
  - cardiovascular disease
  - chronic kidney disease
  - chronic liver disease
  - chronic obstructive pulmonary disease
  - dementia
  - epilepsy
  - gastro-intestinal conditions likely to result in malabsorption (i.e. Crohn’s disease, ulcerative colitis, celiac disease, steatorrhea, blind loop syndrome)
  - meningitis
  - menopausal symptoms
  - Parkinson's disease
  - rheumatoid arthritis
  - systemic lupus erythematosus
  - type 2 diabetes
  - vitamin D deficiency
- Individual medications in the last year
  - anticonvulsants
  - tricyclic antidepressants
  - other antidepressants
  - beta-blockers
  - calcium
  - calcium channel blockers
  - heparin
  - hormone replacement therapy
  - nitrates
  - proton-pump inhibitors
  - steroids
  - vitamin D

Supplementary Table S1. CPRD READ code list for hearing loss/impairment

| **Medcode** | **Readcode** | **Desc** |
| --- | --- | --- |
| 412 | F59..00 | hearing loss |
| 467 | F59..11 | deafness |
| 536 | F591.00 | sensorineural hearing loss |
| 686 | F59z.00 | deafness nos |
| 1151 | F590000 | unspecified conductive hearing loss |
| 1681 | 1C13100 | unilateral deafness |
| 1752 | F580100 | presbyacusis |
| 1774 | 1C13200 | partial deafness |
| 2061 | F591000 | unspecified perceptive hearing loss |
| 2348 | F591.13 | perceptive deafness |
| 2625 | F591.11 | high frequency deafness |
| 3171 | F591211 | nerve deafness |
| 3747 | F593.00 | deaf mutism, nec |
| 4035 | 1C13300 | bilateral deafness |
| 6641 | 1C13.11 | deafness symptom |
| 6846 | F591200 | neural hearing loss |
| 7301 | F592100 | mixed conductive and sensorineural hearing loss, bilateral |
| 7891 | ZE87.17 | hoh - hard of hearing |
| 8033 | F591.12 | low frequency deafness |
| 8941 | F590.00 | conductive hearing loss |
| 9830 | ZE87.00 | hearing loss |
| 9882 | F592.11 | mixed hearing loss |
| 10112 | F591600 | sensorineural hearing loss, bilateral |
| 10367 | ZE87.11 | deafness |
| 10665 | F591400 | congenital sensorineural deafness |
| 12324 | ZE87.16 | hl - hearing loss |
| 12692 | ZE87.15 | hi - hearing impairment |
| 12829 | F591.14 | perceptive hearing loss |
| 14805 | 1C13.00 | deafness |
| 14983 | F592.00 | mixed conductive and sensorineural deafness |
| 16393 | F591z00 | perceptive hearing loss nos |
| 16648 | F59z.11 | chronic deafness |
| 18008 | ZE87.18 | hearing impairment |
| 18520 | F591100 | sensory hearing loss |
| 18945 | F590500 | conductive hearing loss, bilateral |
| 19068 | ZE87.19 | hearing impaired |
| 19084 | F594.00 | high frequency deafness |
| 19666 | ZE87.12 | difficulty hearing |
| 22102 | ZE87.13 | hard of hearing |
| 27901 | F592000 | mix cond/sensneurl hear loss,unlat unrestrc hear/contrlat sd |
| 29191 | F591700 | sensorineurl hear loss,unilat unrestrict hear/contralat side |
| 30033 | ZE87.20 | hearing impaired |
| 30208 | F580111 | senile presbyacusis |
| 30412 | ZE87.14 | hypoacusis |
| 30726 | ZE87100 | dead ear |
| 31748 | F590300 | conductive hearing loss due to disorder of middle ear |
| 33583 | F590z00 | conductive hearing loss nos |
| 36660 | F590100 | conductive hearing loss due to disorder of external ear |
| 38563 | F59y.00 | other specified forms of hearing loss |
| 42720 | F580200 | transient ischaemic deafness |
| 42810 | F580.00 | degenerative and vascular disorders of ear |
| 43581 | F590600 | conduct hear loss,unilat+unrestric hearing on contralat side |
| 44282 | F595.00 | low frequency deafness |
| 44633 | F580000 | unspecified degenerative and vascular disorders of ear |
| 45751 | F590.11 | conductive deafness |
| 47440 | F591500 | ototoxicity - deafness |
| 52758 | F590200 | conductive hearing loss due to disorder of tympanic membrane |
| 54116 | F591y00 | combined perceptive hearing loss |
| 62906 | F590y00 | combined conductive hearing loss |
| 69894 | F591300 | central hearing loss |
| 94392 | F580z00 | degenerative and vascular disorders of ear nos |
| 94782 | F590400 | conductive hearing loss due to disorder of inner ear |
| 98253 | F596.00 | maternally inherited deafness |
| 99753 | F597.00 | mild acquired hearing loss |
| 100127 | F598.00 | moderate acquired hearing loss |
| 100276 | F599.00 | severe acquired hearing loss |
| 100654 | F59A.00 | profound acquired hearing loss |
| 100736 | F591800 | congenital prelingual deafness |
| 102069 | F591511 | drug ototoxicity - deafness |
| 102872 | F59A.11 | deafened |
| 107265 | F591D00 | mild sensorineural hearing loss |
| 107323 | F591A00 | bilateral congenital sensorineural hearing loss |
| 107350 | F591C00 | moderate sensorineural hearing loss |
| 107364 | F591900 | bilateral profound sensorineural hearing loss |
| 107607 | F591B00 | profound sensorineural hearing loss |
| 107610 | F591E00 | severe sensorineural hearing loss |
| 49426 | 7P12.00 | Diagnostic audiology |
| 95102 | 7P12y00 | Other specified diagnostic audiology |
| 49673 | 7P12z00 | Diagnostic audiology NOS |
| 2064 | 8H7V.00 | Refer to audiologist |
| 10479 | 8HT2.00 | Referral to hearing aid clinic |
| 11502 | 8HT3.00 | Referral to audiology clinic |
| 6172 | 9N0W.00 | Seen in audiology clinic |
| 401 | 9N0b.00 | Seen in hearing aid clinic |
| 1827 | 9N2T.00 | Seen by audiologist |
| 90604 | 9b96.00 | Audiological medicine |
| 10329 | ZE...12 | Audiological observations |
| 10037 | ZF...00 | Audiological test observations |
| 32965 | ZL18100 | Under care of audiological physician |
| 30181 | ZL42.00 | Under care of audiologist |
| 46586 | ZL43.00 | Under care of audiology technician |
| 11195 | ZL71.00 | Referral to audiologist |
| 10165 | ZL71.11 | Refer to audiologist |
| 21960 | ZL71100 | Referral to audiological scientist |
| 33260 | ZL71200 | Referral to audiological physician |
| 12093 | ZL71300 | Referral to community doctor in audiology |
| 12305 | ZL71400 | Referral to medical technical officer in audiology |
| 13659 | ZL71500 | Referral to hearing therapist |
| 22831 | ZL71600 | Referral to registered hearing aid dispenser |
| 11925 | ZL71700 | Referral to paediatric audiologist |
| 32773 | ZL9A100 | Seen by audiological physician |
| 11084 | ZLC2.00 | Seen by audiology technician |
| 10938 | ZLD3100 | Discharge by audiological physician |
| 43414 | ZLDE.00 | Discharge by audiologist |
| 32692 | ZLDF.00 | Discharge by audiology technician |
| 32967 | ZLE6200 | Discharge from audiology service |

Supplementary Table S2. Sociodemographic and clinical characteristics of the participants in the study

|  | **HL**  **N=237, 297** | **No HL**  **N=829, 431** |
| --- | --- | --- |
| **Ethnicity**, n (%) |  |  |
| White | 136,196 (57.4) | 441,416 (53.2) |
| Black | 606 (0.3) | 3304 (0.4) |
| Asian | 2266 (1.0) | 7664 (0.9) |
| Other | 878 (0.4) | 3913 (0.5) |
| Not recorded | 97,351 (41.0) | 373,134 (45.0) |
| **Index of multiple deprivation** (quintile), , n (%) |  |  |
| 1 (most affluent) | 27,647 (11.7) | 94,010 (11.3) |
| 2 | 27,571 (11.6) | 93,502 (11.3) |
| 3 | 25,068 (10.6) | 86,848 (10.6) |
| 4 | 18,886 (8.0) | 66,819 (8.1) |
| 5 (most deprived) | 15,125 (6.4) | 52,962 (6.4) |
| **In Care home**, n (%) | 1159 (0.5) | 7147 (0.9) |
| **Smoking status**, n (%) |  |  |
| None | 125,264 (52.8) | 416,814 (50.3) |
| Ex | 82,873 (34.9) | 254,432 (30.7) |
| Light | 13,112 (5.5) | 54,366 (6.6) |
| Moderate | 6859 (2.9) | 30,266 (3.6) |
| Heavy | 4503 (1.9) | 21,398 (2.6) |
| Not recorded | 4686 (2.0) | 52,155 (6.3) |
| **Alcohol consumption**, n (%) |  |  |
| None | 65,952 (27.8) | 213,394 (25.7) |
| Previous | 6567 (2.8) | 22,041 (2.7) |
| Trivial | 66,401 (28.0) | 206,951 (25.0) |
| Light | 40,351 (17.0) | 132,711 (16.0) |
| Moderate | 24,195 (10.2) | 82,750 (10.0) |
| Heavy | 6002 (2.5) | 22,395 (2.7) |
| Very heavy | 3349 (1.4) | 12,936 (1.6) |
| Not recorded | 24,480 (10.3) | 136,253 (16.4) |
| **Body mass index**, n (%) |  |  |
| Mean (SD) | 27.1 (4.7) | 27.3 (5.0) |
| Median (Q1-Q3) | 26.5 (23.8-29.6) | 26.6 (23.8-29.8) |
| 15 to 24 kg/m^2^ | 77,966 (32.9) | 250,329 (30.2) |
| 25 to 29 kg/m^2^ | 89,090 (37.5) | 282,750 (34.1) |
| ≥30 kg/m^2^ | 47,837 (20.2) | 165,634 (20.0) |
| Not recorded | 22,404 (9.4) | 130,718 (15.8) |
| **Morbidities**, n (%) |  |  |
| Anxiety | 29,516 (12.4) | 91,820 (11.1) |
| Asthma | 26,732 (11.3) | 79,456 (9.6) |
| Cancer | 27,828 (11.7) | 84,536 (10.2) |
| Cardiovascular disease | 66,007 (27.8) | 199,294 (24.0) |
| Chronic kidney disease | 2078 (0.9) | 6741 (0.8) |
| Chronic liver disease | 1803 (0.8) | 6024 (0.7) |
| Chronic obstructive pulmonary disease | 17,199 (7.2) | 55,422 (6.7) |
| Dementia | 4809 (2.0) | 26,979 (3.3) |
| Epilepsy | 4073 (1.7) | 12,918 (1.6) |
| Gastro-intestinal disorders | 4138 (1.7) | 11,263 (1.4) |
| Meningitis | 479 (0.2) | 1479 (0.2) |
| Menopausal symptoms in women | 26,403 (23.3) | 79,347 (20.5) |
| Parkinson's disease | 1916 (0.8) | 8925 (1.1) |
| Rheumatoid arthritis | 5043 (2.1) | 15,518 (1.9) |
| Systemic lupus erythematosus | 239 (0.1) | 677 (0.1) |
| Type 2 diabetes | 30,046 (12.7) | 98,510 (11.9) |
| Vitamin D deficiency | 962 (0.4) | 2581 (0.3) |
| **Medications in the last year**, n (%) |  |  |
| Anticonvulsants | 14,206 (6.0) | 41,478 (5.0) |
| Antidepressants, TCA | 19,041 (8.0) | 54,621 (6.6) |
| Antidepressants, other | 24,802 (10.5) | 72,619 (8.8) |
| Beta-blockers | 51,291 (21.6) | 162,002 (19.5) |
| Calcium | 8749 (3.7) | 24,104 (2.9) |
| Calcium channel blockers | 57,087 (24.1) | 177,527 (21.4) |
| Heparin | 362 (0.2) | 1051 (0.1) |
| Hormone replacement therapy in women | 7996 (7.1) | 22,000 (5.7) |
| Nitrates | 21,483 (9.1) | 59,340 (7.2) |
| Proton-pump inhibitors | 73,601 (31.0) | 201,089 (24.2) |
| Systemic steroids | 102,736 (43.3) | 266,489 (32.1) |
| Vitamin D | 17,479 (7.4) | 47,316 (5.7) |

Q: quartile; SD: standard deviation; TCA: tricyclic antidepressant

Supplementary Table S3. MOF during follow-up in individuals with and without HL

|  | **HL**  **N=237, 297** | **No HL**  **N=829, 431** |
| --- | --- | --- |
| **Follow-up** (years) |  |  |
| Mean (SD) | 5.1 (3.9) | 4.4 (3.7) |
| Median (Q1-Q3) | 4.2 (1.9-7.4) | 3.4 (1.5-6.4) |
| **Patients with at least one MOF**, n (%) | 24,222 (10.2) | 60,224 (7.3) |
| Femur | 513 (2.1) | 1406 (2.3) |
| Hip | 6410 (26.5) | 16500 (27.4) |
| Pelvic | 937 (3.9) | 2196 (3.6) |
| Shoulder | 975 (4.0) | 2477 (4.1) |
| Spine | 2307 (9.5) | 5103 (8.5) |
| Tibia | 375 (1.5) | 1093 (1.8) |
| Wrist | 4308 (17.8) | 10786 (17.9) |
| Other | 8397 (34.7) | 20663 (34.3) |

HL: hearing loss; MOF: major osteoporotic fracture; Q: quartile; SD: standard deviation

Supplementary Table S4. Odds ratios for the multivariate analysis for 1- and 10-year hip fracture risk

|  | **OR (95% CI)** | |
| --- | --- | --- |
| **Predictor** | **1-year hip fracture** | **10-year hip fracture** |
| Age >89 | 13.28 (9.81, 17.99) | 6.73 (5.91, 7.66) |
| Age 80-89 | 7.89 (6.02, 10.35) | 6.12 (5.54, 6.77) |
| Femur fx in prior 6 months | 6.68 (2.33, 19.11) | 2.37 (1.00, 5.60) |
| Non-OP fx in prior 6 to 12 months | 3.06 (1.60, 5.84) | 1.89 (1.27, 2.81) |
| Tibia fx in prior 6 to 12 months | 2.98 (0.40, 22.43) | 2.13 (0.64, 7.07) |
| Spine fx in prior 6 to 12 months | 2.93 (1.06, 8.09) | 2.04 (1.09, 3.82) |
| Age 70-79 | 2.86 (2.17, 3.78) | 2.89 (2.62, 3.19) |
| Hip fx in prior 6 months | 2.65 (1.68, 4.19) | 1.79 (1.33, 2.42) |
| Parkinson’s Disease | 2.33 (1.63, 3.34) | 1.77 (1.45, 2.17) |
| Femur fx in prior 12 months | 2.32 (1.22, 4.41) | 1.45 (0.98, 2.13) |
| Shoulder fx in prior 12 months | 1.87 (1.02, 3.45) | 1.51 (1.08, 2.10) |
| Crohn’s disease | 1.76 (0.86, 3.59) | 1.12 (0.76, 1.66) |
| Dementia | 1.75 (1.40, 2.18) | 1.04 (0.90, 1.20) |
| Pelvic fx in prior 6 months | 1.73 (0.54, 5.57) | 1.31 (0.60, 2.86) |
| Pelvic fx in prior 12 months | 1.72 (0.91, 3.26) | 1.90 (1.38, 2.62) |
| RA | 1.70 (1.27, 2.26) | 1.43 (1.23, 1.66) |
| Smoker: moderate | 1.68 (1.18, 2.39) | 1.58 (1.35, 1.84) |
| Wrist fx in prior 6 months | 1.64 (0.67, 4.02) | 1.72 (1.13, 2.62) |
| Coeliac | 1.61 (0.79, 3.26) | 0.88 (0.57, 1.37) |
| Hip fx in prior 12 months | 1.61 (1.20, 2.15) | 1.42 (1.21, 1.66) |
| Pelvic fx in prior 6 to 12 months | 1.59 (0.37, 6.87) | 2.09 (0.94, 4.64) |
| Tibia fx in prior 12 months | 1.54 (0.79, 3.00) | 1.50 (1.12, 2.00) |
| Spine fx in prior 6 months | 1.53 (0.48, 4.86) | 1.31 (0.66, 2.59) |
| Falls | 1.51 (1.32, 1.72) | 1.15 (1.08, 1.23) |
| Non-hip fx in prior 12 months | 1.4 (1.15, 1.71) | 1.28 (1.17, 1.40) |
| Wrist fx in prior 12 months | 1.39 (1.10, 1.76) | 1.32 (1.18, 1.47) |
| Smoker: heavy | 1.35 (0.80, 2.30) | 1.54 (1.25, 1.90) |
| Ethnic: White | 1.35 (1.19, 1.54) | 1.10 (1.04, 1.17) |
| Smoker: light | 1.34 (1.02, 1.77) | 1.28 (1.13, 1.45) |
| Chronic kidney disease | 1.33 (0.84, 2.10) | 1.47 (1.18, 1.83) |
| OP | 1.33 (1.11, 1.59) | 1.12 (1.02, 1.23) |
| Shoulder fx in prior 6 months | 1.28 (0.17, 9.38) | 1.69 (0.67, 4.24) |
| Spine fx in prior 12 months | 1.28 (0.76, 2.18) | 1.32 (1.01, 1.73) |
| COPD | 1.26 (1.02, 1.56) | 1.09 (0.98, 1.21) |
| Chronic liver disease | 1.26 (0.64, 2.46) | 1.04 (0.75, 1.45) |
| Non-OP fx in prior 12 months | 1.23 (0.98, 1.54) | 1.13 (1.02, 1.25) |
| CVD | 1.22 (1.06, 1.40) | 1.07 (1.00, 1.14) |
| SLE | 1.20 (0.29, 5.00) | 1.49 (0.78, 2.85) |
| Charlson_cat2 | 1.19 (0.97, 1.47) | 1.11 (1.01, 1.22) |
| Severe fragility (eFI) | 1.17 (0.81, 1.69) | 0.95 (0.76, 1.20) |
| Difficulty walking | 1.15 (0.91, 1.47) | 0.94 (0.82, 1.08) |
| Charlson_cat3 | 1.14 (0.86, 1.51) | 1.03 (0.90, 1.18) |
| Colitis | 1.09 (0.63, 1.92) | 0.99 (0.75, 1.30) |
| Wrist fx in prior 6 to 12 months | 1.09 (0.34, 3.44) | 0.98 (0.55, 1.75) |
| Alcohol: moderate | 1.07 (0.73, 1.56) | 0.99 (0.83, 1.18) |
| Smoker: none | 1.07 (0.93, 1.24) | 1.06 (0.99, 1.13) |
| Cancer | 1.07 (0.90, 1.28) | 0.94 (0.87, 1.03) |
| Ethnic: Black | 1.07 (0.26, 4.34) | 0.31 (0.12, 0.84) |
| Charlson_cat1 | 1.07 (0.91, 1.27) | 1.09 (1.02, 1.17) |
| Heparin | 1.05 (0.75, 1.45) | 1.03 (0.87, 1.21) |
| Epilepsy | 1.04 (0.68, 1.60) | 1.20 (0.98, 1.47) |
| Non-hip fx in prior 6 to 12 months | 1.03 (0.45, 2.32) | 1.39 (0.97, 1.98) |
| Mild fragility (eFI) | 1.02 (0.89, 1.16) | 1.03 (0.97, 1.10) |
| Anticonvulsive | 1.02 (1.01, 1.03) | 1.00 (1.00, 1.01) |
| Alcohol: trivial | 1.01 (0.73, 1.40) | 0.94 (0.80, 1.11) |
| Proton-pump inhibitors | 1.01 (1.00, 1.01) | 1.00 (0.99, 1.00) |
| Calcium channel blocker | 1.01 (1.00, 1.02) | 1.01 (1.00, 1.01) |
| Nitrates | 1.01 (1.00, 1.02) | 1.01 (1.00, 1.02) |
| Other antidepressant | 1.01 (1.00, 1.02) | 1.01 (1.00, 1.01) |
| Steroids | 1.01 (1.00, 1.02) | 1.01 (1.00, 1.01) |
| Vitamin D deficiency | 1.01 (0.47, 2.16) | 0.63 (0.39, 1.02) |
| Non-hip fx in prior 6 months | 1.01 (0.48, 2.15) | 1.02, 0.70, 1.49) |
| Betablocker | 1.00 (0.99, 1.01) | 0.99 (0.99, 1.00) |
| Calcium | 1.00 (0.98, 1.02) | 0.99 (0.98, 1.00) |
| TCA | 1.00 (0.99, 1.02) | 1.00 (0.99, 1.01) |
| Bisphosphonate | 0.99 (0.97, 1.00) | 0.99 (0.98, 1.00) |
| Vitamin D | 0.98 (0.97, 1.00) | 0.97 (0.96, 0.98) |
| Alcohol: none | 0.97 (0.71, 1.34) | 1.03 (0.88, 1.21) |
| Menopause | 0.97 (0.80, 1.17) | 0.83 (0.76, 0.91) |
| Moderate fragility (eFI) | 0.96 (0.77, 1.18) | 0.96 (0.86, 1.07) |
| Asthma | 0.96 (0.79, 1.17) | 1.01 (0.92, 1.11) |
| HRT | 0.92 (0.83, 1.02) | 0.94 (0.90, 0.98) |
| Care | 0.92 (0.60, 1.41) | 0.66 (0.49, 0.90) |
| Alcohol: very heavy | 0.91 (0.43, 1.94) | 0.81 (0.56, 1.16) |
| Non-OP fx in prior 6 months | 0.91 (0.34, 2.45) | 1.10 (0.71, 1.69) |
| Anxiety | 0.89 (0.74, 1.07) | 0.98 (0.91, 1.07) |
| Hip fx in prior 6 to 12 months | 0.88 (0.32, 2.38) | 1.32 (0.84, 2.07) |
| Alcohol: light | 0.86 (0.60, 1.23) | 0.86 (0.72, 1.02) |
| T2DM | 0.84 (0.68, 1.04) | 0.97 (0.88, 1.06) |
| Family history of OP | 0.81 (0.33, 1.96) | 0.59 (0.37, 0.94) |
| Ethnic: Asian | 0.61 (0.23, 1.66) | 0.49 (0.32, 0.75) |
| Alcohol: heavy | 0.59 (0.29, 1.20) | 0.82 (0.63, 1.08) |
| Sex: male | 0.56 (0.48, 0.65) | 0.55 (0.52, 0.59) |
| BMI 18.6-24.9 | 0.43 (0.29, 0.62) | 0.63 (0.50, 0.78) |
| Ethnic: Other | 0.34 (0.05, 2.44) | 0.80 (0.48, 1.34) |
| BMI 25-29.9 | 0.29 (0.20, 0.42) | 0.47 (0.37, 0.58) |
| BMI 30-39.9 | 0.20 (0.13, 0.31) | 0.35 (0.28, 0.44) |
| BMI ≥40 | 0.08 (0.02, 0.32) | 0.18 (0.11, 0.28) |
| Blind loop syndrome | 0.00 (0.00, inf) | 4.05 (0.52, 31.62) |
| Meningitis | 0.00 (0.00, 3.35E+135) | 1.15 (0.62, 2.10) |
| Steatorrhoea | 0.00 (0.00, inf) | 1.13 (0.27, 4.78) |
| Tibia fx in prior 6 months | 0.00 (0.00, inf) | 2.71 (0.62, 11.8) |
| Femur fx in prior 6 to 12 months | 0.00 (0.00, inf) | 0.74 (0.10, 5.43) |
| Shoulder fx in prior 6 to 12 months | 0.00 (0.00, inf) | 1.32 (0.41, 4.30) |

BMI: body mass index; CI: confidence interval; CVD: cardiovascular disease; fx: fracture; OP: osteoporosis; TCA: tricyclic antidepressant; T2DM: type-2 diabetes mellitus

Supplementary Table S5. Odds ratios for the multivariate analysis for 1- and 10-year MOF risk

|  | **OR (95% CI)** | |
| --- | --- | --- |
| **Predictor** | **1-year MOF** | **10-year MOF** |
| Blind loop syndrome | 3.71 (0.47, 29.46) | 3.42 (1.07, 10.98) |
| Femur fx in prior 6 months | 3.42 (1.45, 8.09) | 2.1 (1.16,3.81) |
| Tibia fx in prior 6 months | 3.23 (0.74, 14.02) | 2.56 (0.99, 6.58) |
| Shoulder fx in prior 6 to 12 months | 3.1 (1.22,7.89) | 2.93 (1.67, 5.14) |
| Hip fx in prior 6 months | 2.99 (2.23, 4.01) | 1.61 (1.30, 2.00) |
| Wrist fx in prior 6 months | 2.9 (1.96, 4.3) | 2.06 (1.61, 2.62) |
| Age >89 | 2.85 (2.51, 3.25) | 1.75 (1.63, 1.88) |
| Shoulder fx in prior 6 months | 2.85 (1.22, 6.67) | 1.92 (1.09, 3.37) |
| Spine fx in prior 6 months | 2.74 (1.57, 4.8) | 2.20 (1.53, 3.16) |
| Non-OP fx in prior 6 months | 2.72 (1.95, 3.81) | 1.7 (1.38, 2.09) |
| Non-hip fx in prior 6 to 12 months | 2.72 (2.01, 3.69) | 2.27 (1.90, 2.72) |
| Pelvic fx in prior 6 months | 2.23 (1.11, 4.48) | 1.97 (1.24, 3.14) |
| Spine fx in prior 6 to 12 months | 2.19 (1.13, 4.21) | 2.22 (1.51, 3.26) |
| Age 80-89 | 2.14 (1.95, 2.36) | 2.05 (1.96, 2.14) |
| Non-hip fx in prior 6 months | 2.06 (1.51, 2.81) | 1.78 (1.49, 2.12) |
| Femur fx in prior 6 to 12 months | 2.05 (0.48, 8.71) | 1.25 (0.48, 3.28) |
| Tibia fx in prior 6 to 12 months | 1.95 (0.46, 8.24) | 1.87 (0.84, 4.14) |
| Hip fx in prior 12 months | 1.86 (1.58, 2.20) | 1.67 (1.52, 1.84) |
| Non-OP fx in prior 6 to 12 months | 1.85 (1.18, 2.88) | 1.78 (1.41, 2.24) |
| Spine fx in prior 12 months | 1.82 (1.41, 2.35) | 1.65 (1.43, 1.90) |
| Wrist fx in prior 12 months | 1.64 (1.46, 1.84) | 1.60 (1.51, 1.70) |
| Non-hip fx in prior 12 months | 1.61 (1.46, 1.78) | 1.46 (1.39, 1.53) |
| Pelvic fx in prior 12 months | 1.61 (1.08, 2.39) | 1.64 (1.33, 2.03) |
| Shoulder fx in prior 12 months | 1.61 (1.1, 2.35) | 1.53 (1.25, 1.86) |
| Chronic liver disease | 1.58 (1.19, 2.10) | 1.36 (1.17, 1.58) |
| Parkinson’s Disease | 1.58 (1.24, 2.01) | 1.38 (1.20, 1.57) |
| OP | 1.44 (1.31, 1.59) | 1.39 (1.32, 1.46) |
| Wrist fx in prior 6 to 12 months | 1.44 (0.82, 2.52) | 1.48 (1.12, 1.96) |
| Femur fx in prior 12 months | 1.44 (0.91, 2.26) | 1.92 (1.56, 2.38) |
| Tibia fx in prior 12 months | 1.43 (1.01, 2.01) | 1.41 (1.20, 1.65) |
| Epilepsy | 1.42 (1.17, 1.74) | 1.36 (1.23, 1.51) |
| Pelvic fx in prior 6 to 12 months | 1.40 (0.50, 3.91) | 1.78 (1.00, 3.15) |
| Hip fx in prior 6 to 12 months | 1.33 (0.8, 2.22) | 1.88 (1.45, 2.43) |
| Non-OP fx in prior 12 months | 1.33 (1.19, 1.49) | 1.36 (1.29, 1.43) |
| Falls | 1.31 (1.22, 1.41) | 1.17 (1.13, 1.22) |
| Age 70-79 | 1.3 (1.19, 1.43) | 1.49 (1.43, 1.54) |
| Severe fragility (eFI) | 1.27 (1.02, 1.57) | 1.00 (0.87, 1.14) |
| Smoker: heavy | 1.26 (1.00, 1.58) | 1.20 (1.08, 1.34) |
| Alcohol: heavy | 1.22 (0.94, 1.57) | 1.10 (0.96, 1.26) |
| Coeliac | 1.21 (0.82, 1.79) | 1.09 (0.89, 1.33) |
| Dementia | 1.21 (1.04, 1.41) | 0.77 (0.70, 0.85) |
| Crohn’s disease | 1.19 (0.78, 1.81) | 1.06 (0.86, 1.31) |
| Smoker: light | 1.16 (1.01, 1.34) | 1.06 (0.99, 1.13) |
| Charlson_cat2 | 1.16 (1.04, 1.29) | 1.02 (0.97, 1.08) |
| COPD | 1.15 (1.03, 1.29) | 1.00 (0.94, 1.06) |
| RA | 1.15 (0.97, 1.37) | 1.24 (1.14, 1.35) |
| Charlson_cat3 | 1.13 (0.97, 1.31) | 0.93 (0.86, 1.01) |
| CVD | 1.11 (1.03, 1.20) | 1.07 (1.03, 1.11) |
| Chronic kidney disease | 1.11 (0.84, 1.46) | 1.09 (0.94, 1.26) |
| Alcohol: moderate | 1.10 (0.90, 1.35) | 1.15 (1.05, 1.27) |
| Mild fragility (eFI) | 1.08 (1.00, 1.16) | 1.07 (1.04, 1.11) |
| Difficulty walking | 1.08 (0.93, 1.25) | 0.82 (0.75, 0.90) |
| Moderate fragility (eFI) | 1.07 (0.96, 1.21) | 1.03 (0.97, 1.09) |
| Ethnic: White | 1.07 (1.01, 1.14) | 0.90 (0.87, 0.93) |
| Charlson_cat1 | 1.07 (0.99, 1.17) | 1.03 (0.99, 1.06) |
| Smoker: moderate | 1.06 (0.88, 1.29) | 1.11 (1.02, 1.21) |
| Alcohol: light | 1.05 (0.87, 1.25) | 1.03 (0.94, 1.12) |
| Cancer | 1.05 (0.96, 1.16) | 0.98 (0.94, 1.03) |
| Alcohol: very heavy | 1.04 (0.74, 1.46) | 1.04 (0.88, 1.23) |
| Asthma | 1.03 (0.93, 1.14) | 1.06 (1.01, 1.11) |
| Family history of OP | 1.02 (0.71, 1.46) | 0.88 (0.73, 1.05) |
| Menopause | 1.02 (0.93, 1.12) | 0.93 (0.89, 0.97) |
| Anticonvulsive | 1.01 (1.00, 1.02) | 1.01 (1.00, 1.01) |
| Nitrates | 1.01 (1.00, 1.01) | 1.01 (1.00, 1.01) |
| Other antidepressant | 1.01 (1.01, 1.02) | 1.01 (1.00, 1.01) |
| Steroids | 1.01 (1.00, 1.01) | 1.00 (1.00, 1.01) |
| TCA | 1.01 (1.00, 1.01) | 1.00 (1.00, 1.01) |
| Proton-pump inhibitors | 1.00 (1.00, 1.01) | 1.00 (1.00, 1.00) |
| Betablocker | 1 (0.99, 1.00) | 0.99 (0.99, 1.00) |
| Calcium | 1 (0.99, 1.01) | 1.00 (0.99, 1.00) |
| Calcium channel-blocker | 1.00 (1.00, 1.01) | 1.00 (1.00, 1.00) |
| Vitamin D | 1 (0.99, 1.00) | 0.98 (0.97, 0.98) |
| Vitamin D deficiency | 1.00 (0.67, 1.49) | 0.62 (0.48, 0.80) |
| Smoker: none | 0.99 (0.92, 1.06) | 1.01 (0.98, 1.05) |
| Bisphosphonate | 0.99 (0.99, 1.00) | 1.01 (1.00, 1.01) |
| Anxiety | 0.99 (0.91, 1.08) | 1.01 (0.97, 1.05) |
| Heparin | 0.96 (0.75, 1.22) | 1.00 (0.91, 1.10) |
| T2DM | 0.96 (0.86, 1.06) | 0.97 (0.93, 1.03) |
| Alcohol: none | 0.94 (0.79, 1.11) | 1.04 (0.96, 1.14) |
| HRT | 0.93 (0.90, 0.98) | 0.97 (0.95, 0.98) |
| Alcohol: trivial | 0.92 (0.77, 1.09) | 0.97 (0.89, 1.06) |
| Colitis | 0.84 (0.61, 1.17) | 1.01 (0.87, 1.16) |
| Care | 0.81 (0.61, 1.09) | 0.57 (0.47, 0.71) |
| Ethnic: Asian | 0.75 (0.50, 1.12) | 0.50 (0.41, 0.61) |
| Ethnic: Other | 0.73 (0.39, 1.37) | 0.71 (0.55, 0.93) |
| Meningitis | 0.68 (0.30, 1.52) | 1.52 (1.15, 2.01) |
| BMI 18.6-24.9 | 0.6 (0.46, 0.77) | 0.72 (0.62, 0.83) |
| Ethnic: Black | 0.55 (0.23, 1.34) | 0.34 (0.22, 0.54) |
| Steatorrhoea | 0.48 (0.07, 3.58) | 0.90 (0.42, 1.93) |
| BMI 25-29.9 | 0.48 (0.37, 0.62) | 0.60 (0.51, 0.70) |
| Sex: male | 0.45 (0.42, 0.49) | 0.43 (0.42, 0.45) |
| SLE | 0.43 (0.14, 1.35) | 0.96 (0.64, 1.43) |
| BMI 30-39.9 | 0.39 (0.29, 0.50) | 0.49 (0.42, 0.58) |
| BMI ≥40 | 0.34 (0.22, 0.50) | 0.34 (0.31, 0.49) |

BMI: body mass index; CI: confidence interval; CVD: cardiovascular disease; fx: fracture; OP: osteoporosis; TCA: tricyclic antidepressant; T2DM: type-2 diabetes mellitus

Supplementary Table S6. Logistic regression model estimates for 1-year hip and MOF risk in patients with HL

|  | **Hip fracture** | | **MOF** | |
| --- | --- | --- | --- | --- |
|  | **OR** | **p-value** | **OR** | **p-value** |
| Age >89 years | 2.63 | <0.0001 | 1.06 | <0.0001 |
| Age 80-89 years | 2.08 | <0.0001 | 0.75 | <0.0001 |
| Age 70-79 years | 1.09 | <0.0001 | 0.25 | <0.0001 |
| Femur fx in prior 12 months | 0.77 | 0.03 | 0.29 | 0.2 |
| Shoulder fx in prior 12 months | 0.61 | 0.06 | 0.46 | 0.02 |
| Pelvic fx in prior 12 months | 0.51 | 0.14 | 0.48 | 0.019 |
| Tibia fx in prior 12 months | 0.48 | 0.16 | 0.35 | 0.0499 |
| Hip fx in prior 12 months | 0.44 | 0.003 | 0.52 | <0.0001 |
| Falls | 0.41 | <0.0001 | 0.34 | <0.0001 |
| Non-hip fx in prior 12 months | 0.33 | 0.002 | 0.42 | <0.0001 |
| Ethnic: White | 0.33 | <0.0001 | 0.07 | 0.04 |
| Smoker: heavy | - | - | 0.31 | 0.01 |
| Spine fx in prior 12 months | 0.30 | 0.26 | 0.55 | <0.0001 |
| Osteoporosis | 0.29 | 0.0009 | 0.41 | <0.0001 |
| Severe fragility (eFI) | - | - | 0.28 | 0.02 |
| COPD | 0.26 | 0.02 | 0.13 | 0.03 |
| Charlson_cat2 | 0.25 | 0.01 | 0.15 | 0.004 |
| Non-OP fx in prior 12 months | 0.23 | 0.045 | 0.23 | 0.0001 |
| Wrist fx in prior 12 months | 0.20 | 0.11 | 0.38 | <0.0001 |
| CVD | 0.16 | 0.02 | 0.11 | 0.004 |
| Ethnic: Black | 0.16 | 0.83 | -0.54 | 0.23 |
| Charlson_cat3 | 0.15 | 0.25 | 0.13 | 0.08 |
| Smoker: light | - | - | 0.15 | 0.04 |
| Moderate fragility (eFI) | - | - | 0.11 | 0.08 |
| Smoker: moderate | - | - | 0.10 | 0.32 |
| Mild fragility (eFI) | - | - | 0.10 | 0.008 |
| Cancer | 0.07 | 0.42 | 0.06 | 0.21 |
| Charlson_cat1 | 0.07 | 0.42 | 0.07 | 0.10 |
| Alcohol: moderate | 0.04 | 0.84 | 0.06 | 0.58 |
| Proton-pump inhibitors | 0.01 | 0.02 | 0.01 | 0.0002 |
| Calcium channel blockers | 0.008 | 0.09 | 0.0009 | 0.76 |
| Steroids | 0.01 | 0.047 | 0.006 | 0.06 |
| Smoker: none | - | - | -0.001 | 0.98 |
| Alcohol: none | -0.09 | 0.60 | -0.07 | 0.45 |
| Alcohol: trivial | -0.08 | 0.62 | -0.12 | 0.21 |
| Alcohol: very heavy | -0.16 | 0.70 | 0.14 | 0.32 |
| Alcohol: light | -0.27 | 0.14 | 0.008 | 0.93 |
| Sex: male | -0.58 | <0.0001 | -0.80 | <0.0001 |
| Alcohol: heavy | -0.71 | 0.08 | 0.14 | 0.32 |
| Ethnic: Asian | -0.73 | 0.21 | -0.46 | 0.042 |
| BMI 18.6-24.9 | -0.91 | <0.0001 | -0.56 | <0.0001 |
| Ethnic: other | -0.97 | 0.33 | -0.37 | 0.28 |
| BMI 25-29.9 | -1.35 | <0.0001 | -0.79 | <0.0001 |
| BMI 30-39.9 | -1.71 | <0.0001 | -1.00 | <0.0001 |
| BMI ≥40 | -2.55 | 0.0003 | -1.13 | <0.0001 |
| Intercept | -5.95 | <0.0001 | -3.78 | <0.0001 |

BMI: body mass index; CVD: cardiovascular disease; fx: fracture; OP: osteoporosis;


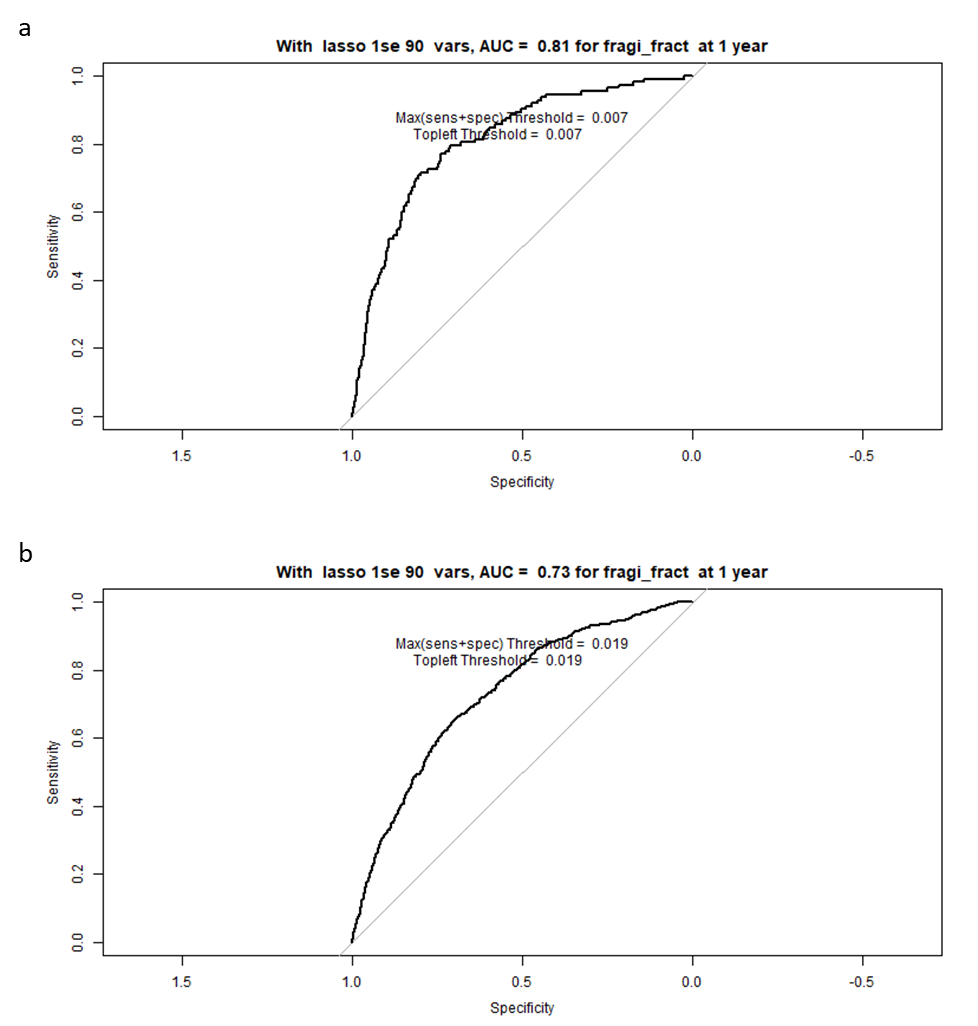


Figure S1. AUC plot the 1-year hip- (a) and MOF models (b)

**REFERENCES**

1. Vergouwe Y, Steyerberg EW, Eijkemans MJ, Habbema JD. Substantial effective sample sizes were required for external validation studies of predictive logistic regression models. J Clin Epidemiol. 2005;58(5):475-83.

2. British Academy of Audiology. Hearing loss and deafness. [Available from: <https://www.baaudiology.org/about/media-centre/facts-about-hearing-loss-and-deafness/>.

3. Royal National Institute for Deaf People. Hearing Matters Report 2020 [Available from: <https://rnid.org.uk/wp-content/uploads/2020/05/Hearing-Matters-Report.pdf>.
